# Prediction performance of the machine learning model in predicting mortality risk in patients with traumatic brain injuries: a systematic review and meta-analysis

**DOI:** 10.1186/s12911-023-02247-8

**Published:** 2023-07-29

**Authors:** Jue Wang, Ming Jing Yin, Han Chun Wen

**Affiliations:** 1 Department of Emergency, The First Affiliated Hospital of Guangxi Medical University, 530021 Nanning, Guangxi China; 2 Intensive Care Department, Guangxi Medical University First Affiliated Hospital, Ward 1, No. 6 Shuangyong Road, Qingxiu District, Guangxi Zhuang Autonomous Region Nanning, China

**Keywords:** Machine learning, Traumatic brain injury, Meta-analysis, Prediction, Mortality rate, Systematic review

## Abstract

**Purpose:**

With the in-depth application of machine learning(ML) in clinical practice, it has been used to predict the mortality risk in patients with traumatic brain injuries(TBI). However, there are disputes over its predictive accuracy. Therefore, we implemented this systematic review and meta-analysis, to explore the predictive value of ML for TBI.

**Methodology:**

We systematically retrieved literature published in PubMed, Embase.com, Cochrane, and Web of Science as of November 27, 2022. The prediction model risk of bias(ROB) assessment tool (PROBAST) was used to assess the ROB of models and the applicability of reviewed questions. The random-effects model was adopted for the meta-analysis of the C-index and accuracy of ML models, and a bivariate mixed-effects model for the meta-analysis of the sensitivity and specificity.

**Result:**

A total of 47 papers were eligible, including 156 model, with 122 newly developed ML models and 34 clinically recommended mature tools. There were 98 ML models predicting the in-hospital mortality in patients with TBI; the pooled C-index, sensitivity, and specificity were 0.86 (95% CI: 0.84, 0.87), 0.79 (95% CI: 0.75, 0.82), and 0.89 (95% CI: 0.86, 0.92), respectively. There were 24 ML models predicting the out-of-hospital mortality; the pooled C-index, sensitivity, and specificity were 0.83 (95% CI: 0.81, 0.85), 0.74 (95% CI: 0.67, 0.81), and 0.75 (95% CI: 0.66, 0.82), respectively. According to multivariate analysis, GCS score, age, CT classification, pupil size/light reflex, glucose, and systolic blood pressure (SBP) exerted the greatest impact on the model performance.

**Conclusion:**

According to the systematic review and meta-analysis, ML models are relatively accurate in predicting the mortality of TBI. A single model often outperforms traditional scoring tools, but the pooled accuracy of models is close to that of traditional scoring tools. The key factors related to model performance include the accepted clinical variables of TBI and the use of CT imaging.

**Supplementary Information:**

The online version contains supplementary material available at 10.1186/s12911-023-02247-8.

## Instruction

Traumatic brain injury (TBI) is a major global health problem, with 950,000 TBI cases per year [1], which is mainly triggered by falls and road injuries. With an increasing population density, the aging of the population, and the use of motor vehicles, motorcycles, and bicycles, the incidence of TBI may continue to increase over time, and patients with TBI are prone to death and disability [2]. Among various injuries, TBI accounts for 1/3 to 1/2 of all deaths from injuries and is the leading cause of disability in patients aged below 40 [3]. TBI has caused a heavy burden in low- and middle-income countries due to the high incidence, mortality, disability, social economic losses, and reduced life expectancy and life quality [4]. Therapeutic treatments are often determined based on the prognosis of patients. Hence, reliable prognosis is essential for a neurosurgeon to decide whether to perform surgical treatment [5]. The use of calculation tools for the predication of TBI patient’s prognosis facilitates physicians to increase or reduce the frequency of interventions in patients with good or poor prognoses respectively.

The scoring tools currently applied in clinical practice to judge the prognosis of TBI patients include the Glasgow Outcome Scale and Head Marshall CT Classification [6, 7], with differences in the use of scoring tools for different wards, for instance, SOFA (Sequential Organ Failure Assessment), APACHE II (Acute Physiology and Chronic Health Disease Classification System II), and GCS (Glasgow Coma Scale) are often used in intensive care units (ICUs) to roughly assess the prognosis of patients [8]. However, diversified evaluation nodes and usage scenarios often make these tools fail to achieve an expected predictive performance in the precise stratification of TBI patients. For example, there are controversies between the GCS score and the Full Outline of Unresponsiveness (FOUR) Score, although the latter was developed to address the evaluation shortcomings of the former [9, 10].

Moreover, there are currently many types of scoring tools available, but neither comprehensive and systematic exploration of the predictive factors related to prognosis, nor horizontal comparison has been reported. Whether there are differences in predicting prognosis between these tools and how to choose an appropriate clinical scoring tool remain to be elucidated. Over the years, some scholars have applied the machine learning(ML) method to establish predictive models for patients with traumatic brain injuries(TBI), including neural network(NN), Naive Bayes(NB), logistic regression(LR), decision tree(DT), and support vector machine(SVM) [11, 12]. ML prediction models differ from previous scoring tools in predictive factors. However, there is a lack of evidence to underpin that the prediction performance of predictive factors in ML models is better than that of traditional scoring tools. Moreover, whether ML prediction models have better predictive performance than clinical scoring tools remains controversial. The latest guidelines for severe brain injury management do not provide an answer to this question. Therefore, we conducted this systematic review and meta-analysis to explore the predictive value of ML versus traditional scoring tools in the mortality risk stratification of TBI patients.

## Methodology

The systematic review was implemented in accordance with the Preferred Reporting Items for Systematic Reviews and Meta-Analyses of Diagnostic Test Accuracy Reviews (PRISMA-DTA) statements. The study protocol has been registered in the International Prospective Register of Systematic Reviews (PROSPERO) and approved prior to the start of the study (ID: CRD42022377756).

### Inclusion and exclusion criteria

#### Inclusion criteria

1. The study subjects are patients diagnosed with TBI;
2. The study types are case-control studies, cohort studies, nested case-control studies, and case-cohort studies;
3. A complete prediction model for mortality risk was constructed;
4. Studies without external verification were also included;
5. Different studies on ML algorithms were published based on the same data set;
6. Studies reported in English were included.

#### Exclusion criteria

1. Studies of the following types: meta-analysis, review, guidance, expert opinion, etc.;
2. Only risk factors were analyzed, and no complete ML model was constructed;
3. Studies lacking the following outcome measures which are used to assess the accuracy of the ML model: Roc, C-statistics, C-index, sensitivity, specificity, accuracy, recovery rate, accuracy rate, confusion matrix, diagnostic four-grid table, F1 score, and calibration curve;
4. Studies on the accuracy of single-factor prediction.

### Retrieval strategy

As of November 27, 2022, a systematic retrieval was conducted in the databases including PubMed, Embase.com, Cochrane, and Web of Science. The retrieval terms include subject terms and free text words. The following terms (including synonyms and closely related words) are used as index terms or free text words: "traumatic brain injuries", and "machine learning". Only peer-reviewed articles are included. The complete retrieval strategy for all databases is provided in Table S1.

### Literature screening and data extraction

We imported the retrieved literature into Endnote, screened the initial eligible original studies by title and abstract after checking for duplication, downloaded the full texts of the potentially eligible studies, and then determined the final eligible original studies according to a full-text review. Before data extraction, a standard data extraction spreadsheet was prepared. The extracted data contained the title of the literature, the author of the literature, year of publication, country of the author, study type, patient source, background of TBI occurred, time of death, number of dead samples, total sample size, number of dead samples in the training set, total sample size in the training set, generation method of the verification set, number of dead samples in the verification set, sample size of the verification set, processing method for missing values, variable screening method, type of model used, and modeling variables. The literature screening and data extraction mentioned above were implemented by two independent investigators (JW and MJY). Dissents, if any, were solved by a third investigator (HCW).

### Risk of bias assessment

Two investigators used the prediction model risk of bias(ROB) assessment tool (PROBAST) to assess the ROB of each model in each study and the applicability of our reviewed questions [13]. Disagreements, if any, were solved by a third investigator. Based on a series of specific questions, each model was respectively assessed as having a "high", "unclear" or "low" ROB in four domains (participators, predictors, outcomes, and analysis). The same scale was used to assess the applicability of each model to our reviewed questions in three domains (participators, predictors, and results).

### Outcome measures

To quantitatively summarize the prediction performance of ML models, we extracted discrimination and calibration measures. The discriminative ability quantified the capacity of the models to distinguish individual development from non-development results. The C-index or the area under the receiver operating characteristic curve (AUROC) with a 95% confidence interval (95% CI) was extracted. When the number of death events was very small in original studies, the C-index was insufficient to reflect the accuracy of models in predicting death cases, so our outcome measures also included sensitivity and specificity. If none of such outcomes were available but the AUC value was reported, sensitivity and specificity were obtained from the Roc curve based on the optimal Youden index.

### Statistical methods

Meta-analysis was performed on the C-index and accuracy of ML models. If the C-index lacks 95% CI and standard errors, we estimated the standard errors by referring to the studies of Debray TP et al. [14]. Given the differences in the variables included in the ML models and the inconsistency in parameters, a random-effects model was preferred for the meta-analysis of the C-index. In addition, a bivariate mixed-effects model was adopted for the meta-analysis of the sensitivity and specificity. The meta-analysis was conducted using R4.2.0 (R development Core Team, Vienna, http://www.R-project.org).

## Result

### Literature screening results

**Fig. 1:**
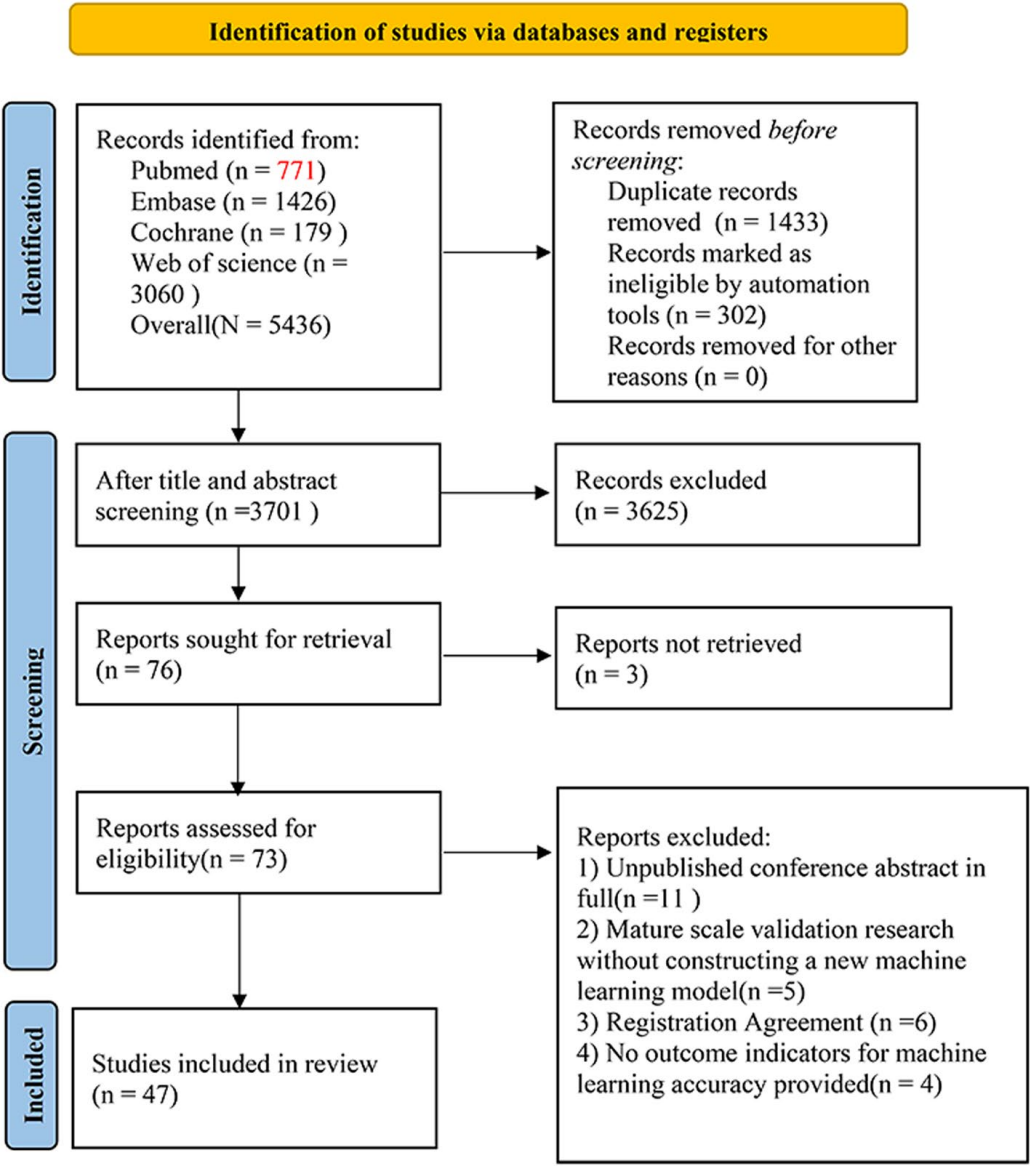
Literature screening process

The study selection process is shown in Fig. 1. A total of 3,701 unique records were identified, and 76 reports were reviewed. After excluding studies with no full texts, conference abstracts, registration protocols, failure to provide any outcome measures for ML accuracy, and failure to create a new ML model, 47 studies were finally included [11, 12, 15–59].

### Basic characteristics of the included literature

The 47 included studies were from Asia (*N* = 17, 36%), Europe (*N* = 16, 34%), America (*N* = 12, 26%), and Middle East (*N* = 2, 4%). The total number of subjects was 2,080,819. The study type included case-control studies (*N* = 32), prospective cohort studies (*N* = 10), and retrospective cohort studies (*N* = 4). Most articles were mainly published in 2022 (*N* = 10), followed by 2020 (*N* = 7), and 2018 (*N* = 6). Totally, 156 models were included, including 122 newly-developed ML models, of which there were 98 ML models for predicting in-hospital mortality, and 24 ML models for predicting out-of-hospital mortality. The ML models for predicting in-hospital mortality mainly included the SVM (*N* = 16), DT (*N* = 13), LR (*N* = 12), random forest(RF) (*N* = 10), K-Nearest Neighbor(KNN) (*N* = 8), and NN (*N* = 8).

### Predictors

In the newly-developed ML models, the most common variable was the GCS score (*N* = 88; 9.6%), age (*N* = 74; 8.1%), CT classification (*N* = 72; 7.9%), pupil size/light reflex (*N* = 31; 3.4%), glucose (*N* = 30; 3.3%), systolic blood pressure (*N* = 30; 3.3%), hypotension (*N* = 15; 1.6%), heart rate (*N* = 15; 1.6%), and hypoxemia: (*N* = 14; 1.5%). The specific predictors are presented in Table S2.

### Risk of bias assessment result

**Fig. 2:**
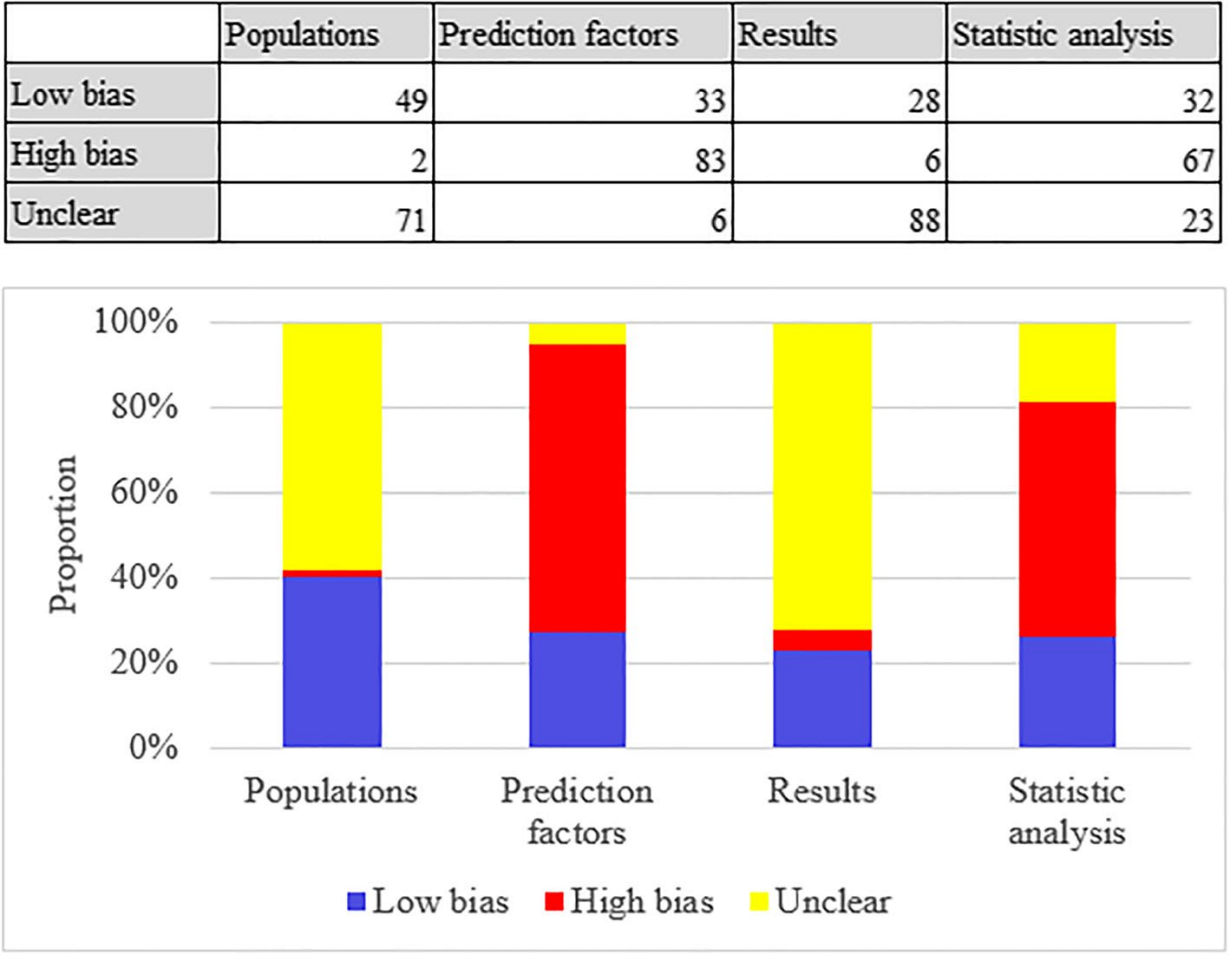
ROB assessment result

Most study subjects were distributed in case-control studies, and the ROB was low in the domain of participants. Regarding the domain of predictors, there was a high bias in whether predictors were assessed without clear result data (*N* = 83; 68%). Because most studies included were case-control studies, and only a small number of them were prospective studies and retrospective cohort studies, the ROB in outcomes was low or unclear. As for the statistical analysis, there was a high bias (*N* = 67) in terms of whether the sample size was reasonable, which was mainly caused by the lack of an independent verification cohort or a sample size that was less than 100 for verification. There was a small amount of high bias in terms of whether the selection of predictors by a single-factor analysis was avoided, while the rest were at a low or unclear ROB (Fig. 2).

### Meta-analysis Result

#### In-hospital mortality

##### Newly-developed machine learning models

**Fig. 3:**
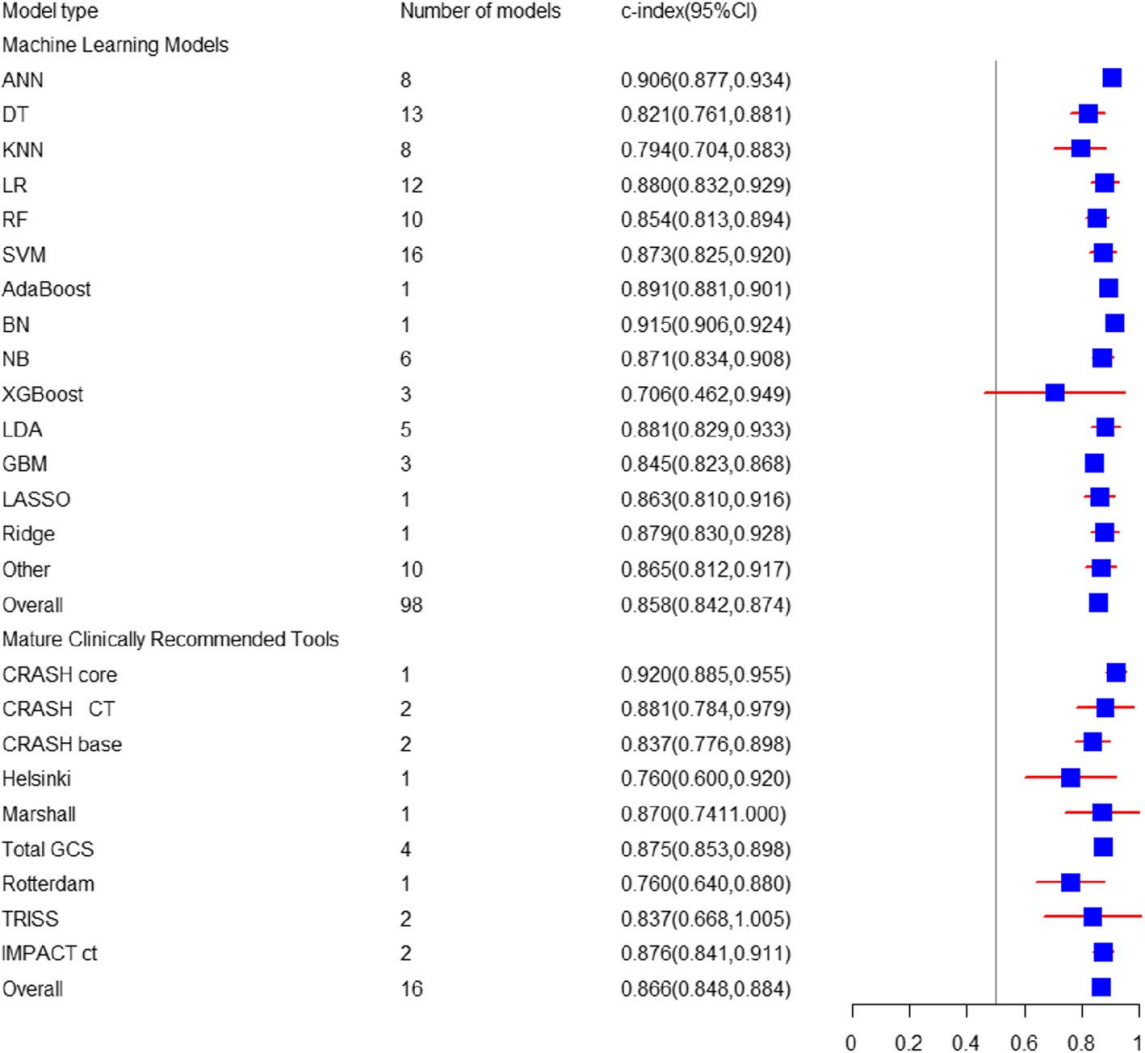
Forest map of c-index prediction of in-hospital deaths by newly developed ML models and clinically recommended tools

The pooled C-index of 98 ML prediction models in predicting in-hospital mortality was 0.86 (95% CI: 0.84, 0.87) in the training set (Fig. 3). The sensitivity and specificity of 64 models were reported, and the pooled sensitivity and specificity were 0.79 (95% CI: 0.75, 0.82) and 0.89 (95% CI: 0.86, 0.92), respectively. For the in-hospital mortality, the verification set only included LR (*N* = 3) [C-index: 0.86 (95% CI: 0.81, 0.90); sensitivity: 0.59 to 0.66; specificity: 0.93 to 0.92]. The most common algorithms in ML prediction included the SVM (*N* = 16) [C-index: 0.87 (95% CI: 0.83, 0.92); sensitivity: 0.86 (95% CI: 0.80, 0.91); specificity: 0.87 (95% CI: 0.74, 0.94)], the DT (*N* = 13) [C-index: 0.82 (95% CI: 0.76, 0.88); sensitivity: 0.83 (95% CI: 0.67, 0.92); specificity: 0.93 (95% CI: 0.78, 0.98)], the LR (*N* = 12) [C-index: 0.88 (95% CI: 0.83, 0.93); sensitivity: 0.68 (95% CI: 0.58, 0.77); specificity: 0.91 (95% CI: 0.85, 0.95)], the RF (*N* = 10) [C-index: 0.85 (95% CI: 0.81, 0.89); sensitivity: 0.86 (95% CI: 0.73, 0.93); specificity: 0.92 (95% CI: 0.76, 0.98)], the NN (*N* = 8) [C-index: 0.91 (95% CI: 0.88, 0.93); sensitivity: 0.78 (95% CI: 0.57, 0.91); specificity: 0.92 (95% CI: 0.87, 0.95)], and the KNN (*N* = 8) [C-index: 0.79 (95% CI: 0.70, 0.88)] (Tables 1 and 2).

**Table 1:** C-index of prediction models for in-hospital and out-of-hospital mortality in the training set and verification set

Subgroup	Model type	Training Cohort		Verification Cohort	
		Number	c-index(95%CI)	Number	c-index(95%CI)
**Death in hospital**	**Newly Developed Machine Learning Models**				
	ANN	8	0.906(0.877,0.934)		
	DT	13	0.821(0.761,0.881)		
	KNN	8	0.794(0.704,0.883)		
	LR	12	0.880(0.832,0.929)	3	0.855(0.806,0.904)
	RF	10	0.854(0.813,0.894)		
	SVM	16	0.873(0.825,0.920)		
	AdaBoost	1	0.891(0.881,0.901)		
	BN	1	0.915(0.906,0.924)		
	NB	6	0.871(0.834,0.908)		
	XGBoost	3	0.706(0.462,0.949)		
	LDA	5	0.881(0.829,0.933)		
	GBM	3	0.845(0.823,0.868)		
	LASSO	1	0.863(0.810,0.916)		
	Ridge	1	0.879(0.830,0.928)		
	Other	10	0.865(0.812,0.917)		
	**Overall**	**98**	**0.858(0.842,0.874)**	**3**	**0.855(0.806,0.904)**
	**Mature Clinically Recommended Tools**				
	CRASH core	1	0.920(0.885,0.955)		
	CRASH CT	2	0.881(0.784,0.979)		
	CRASH base	2	0.837(0.776,0.898)		
	Helsinki	1	0.760(0.600,0.920)	1	0.720(0.430,1.010)
	Marshall	1	0.870(0.7411.000)	1	0.690(0.380,1.000)
	Total GCS	4	0.875(0.853,0.898)		
	Rotterdam	1	0.760(0.640,0.880)	1	0.660(0.410,0.910)
	TRISS	2	0.837(0.668,1.005)		
	IMPACT ct	2	0.876(0.841,0.911)		
	**Overall**	**16**	**0.866(0.848,0.884)**	**3**	**0.687(0.525,0.848)**
Out-of-hospital death	**Newly Developed Machine Learning Models**				
	ANN	2	0.829(0.797,0.861)	1	0.820(0.795,0.845)
	DT	2	0.833(0.666,1.001)		
	ICP-MAP-CPP	3	0.814(0.782,0.845)	2	0.765(0.707,0.823)
	LR	10	0.844(0.807,0.881)	2	0.820(0.807,0.833)
	GBM	1	0.810(0.785,0.835)	1	0.830(0.805,0.855)
	RF	3	0.805(0.741,0.868)	1	0.810(0.780,0.840)
	SVM	2	0.831(0.794,0.868)	1	0.810(0.785,0.835)
	Ridge	1	0.810(0.785,0.835)	1	0.820(0.795,0.845)
	**Overall**	**24**	**0.828(.807,0.849)**	**9**	**0.818(0.809,0.826)**
	**Mature Clinically Recommended Tools**				
	CRASH base	1	0.860(0.820,0.900)		
	CRASH CT	2	0.896(0.828,0.965)		
	CRASH core	1	0.920(0.895,0.945)		
	Hukkelhoven	1	0.784(0.76,0.804)		
	Total GCS	1	0.788(0.747,0.829)		
	APACHE II	1	0.800(0.755,0.845)	1	0.810(0.760,0.860)
	IMPACT extended	4	0.804(0.772,0.837)	1	0.810(0.760,0.860)
	IMPACT lab	3	0.783(0.765,0.801)	1	0.820(0.775,0.865)
	IMPACT core	4	0.782(0.751,0.814)	1	0.810(0.765,0.855)
	**Overall**	18	0.817(0.789,0.845)	4	0.813(0.789,0.836)

**Table 2:** Sensitivity and specificity of prediction models for in-hospital and out-of-hospital mortality in the training set and verification set

Subgroup	Model type	Training Cohort			Verification Cohort		
		Number	Sen(95%CI)	Spe(95%CI)	Number	Sen(95%CI)	Spe(95%CI)
Death in hospital	**Newly Developed Machine Learning Models**						
	ANN	7	0.78(0.57,0.91)	0.92(0.87,0.95)	1	0.84	0.93
	DT	6	0.83(0.67,0.92)	0.93(0.78,0.98)	2	0.44-1	0.98–0.98
	KNN	1	0.91				
	LR	9	0.68(0.58,0.77)	0.91(0.85,0.95)	2	0.59–0.66	0.92–0.94
	RF	7	0.86(0.73,0.93)	0.92(0.76,0.98)			
	SVM	12	0.86(0.80,0.91)	0.87(0.74,0.94)	1	0.66	0.95
	NB	6	0.82(0.74,0.87)	0.89(0.70,0.96)	1	0.59	0.89
	LDA	2	0.66–0.85	0.48–0.81			
	GBM	3	0.76–0.94	0.63–0.81			
	LASSO	1	0.95	0.48			
	Ridge	1	0.88	0.71			
	AdaBoost	1	0.34	0.97			
	xgboost	1	0.79	0.81			
	Other	7	0.67(0.50,0.80)	0.88(0.65,0.97)			
	**Overall**	**64**	**0.79(0.75,0.82)**	**0.89(0.86,0.92)**	**7**	**0.64(0.53,0.75)**	0.95(0.92,0.97)
	**Mature Clinically Recommended Tools**						
	CRASH core	1	0.73	0.90			
	CRASH CT	2	0.72–0.84	0.72–0.90			
	CRASH base	2	0.72–0.78	0.80–0.82			
	Helsinki	1	0.83	0.59	1	0.66	0.84
	Marshall	1	0.75	0.98	1	0.50	0.99
	Total GCS	2	0.50–0.51	0.90–0.90			
	Rotterdam	1	0.58	0.77	1	0.66	0.64
	IMPACT ct	1	0.80	0.78			
	**Overall**	11	0.71(0.64,0.78)	0.84(0.78,0.89)			
Death within 1 month	**Newly Developed Machine Learning Models**						
	DT	1	0.71	0.77			
	LR	2	0.70–0.77	0.61–0.75			
	RF	1	0.80	0.84			
	SVM						
	LASSO/Rige						
	**Overall**	**4**	**0.74(0.67,0.81)**	**0.75(0.66,0.82)**			
	**Mature Clinically Recommended Tools**						
	CRASH core	1	0.77	0.89			
	CRASH CT	1	0.87	0.88			
	Total GCS	1	0.60	0.70			
	APACHE II	1	0.75	0.75			
	IMPACT core	1	0.70	0.74			
	IMPACT extended	1	0.76	0.74			
	IMPACT lab	1	0.72	0.76			
	**Overall**	7	0.75(0.68,0.80)	0.79(0.73,0.84)			

##### Mature Clinically Recommended Tools

**Fig. 4:**
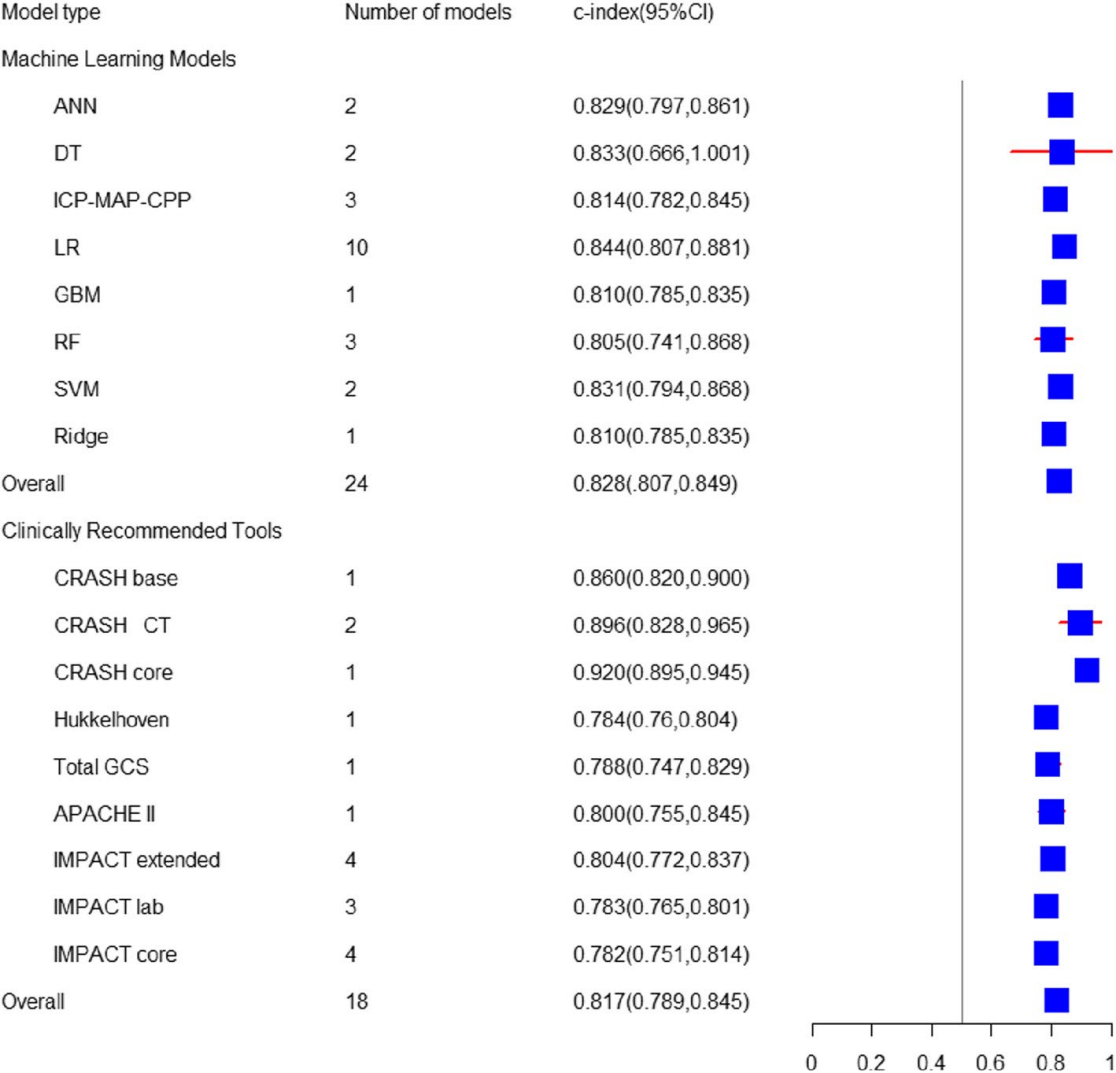
Forest map of c-index prediction of out-of-hospital deaths by newly developed ML models and clinically recommended tools

**Fig. 5:**
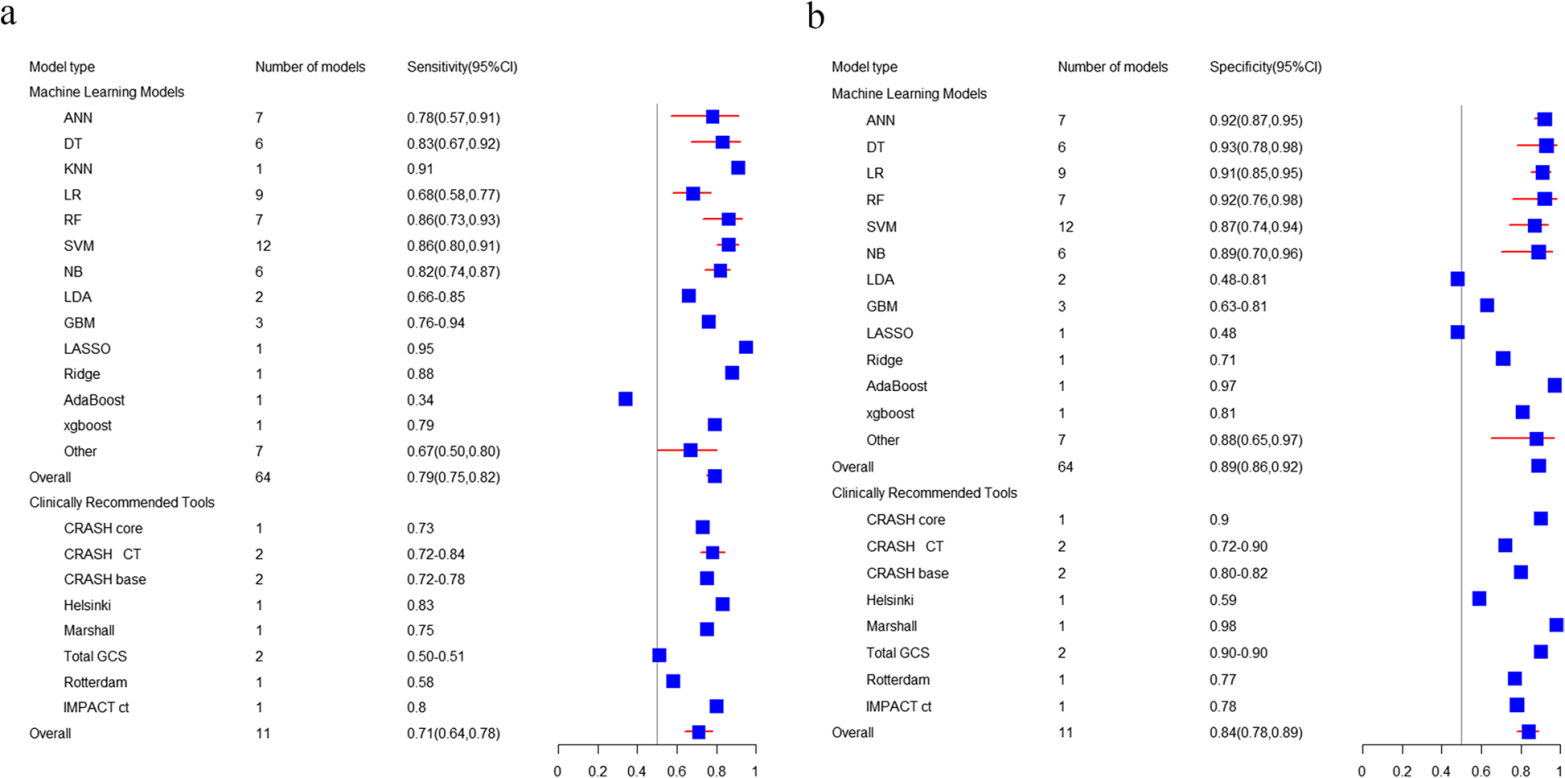
**a **Forest plots of sensitivity of newly developed ML models and clinically recommended tools to predict in-hospital death. **b** Forest plots of specificity of newly developed ML models and clinically recommended tools to predict in-hospital death

There were 16 clinical scoring tools and some tools externally validated in large-scale cohort studies for predicting the in-hospital mortality, with a pooled C-index of 0.86 (95% CI: 0.85, 0.88) in the training set. The sensitivity and specificity of 11 tools were reported, and the pooled sensitivity and specificity were 0.71 (95% CI: 0.64, 0.78) and 0.84 (95% CI: 0.78, 0.89), respectively. In the verification set, there were only 3 tools predicting in-hospital mortality, with a pooled C-index of 0.687 (95% CI: 0.525, 0.848), with no sensitivity or specificity (Figs. 3, 4 and 5a, b).

#### Out-of-hospital mortality

##### Newly developed machine learning models

There were 24 ML prediction models predicting the out-of-hospital mortality in the training set, with a pooled C-index of 0.83 (95%CI: 0.81, 0.85). The sensitivity and specificity 4 models were reported, and the pooled sensitivity and specificity were 0.74 (95%CI: 0.67, 0.81) and 0.75 (95%CI: 0.66, 0.82), respectively. In the verification set, there were 9 prediction models predicting the out-of-hospital mortality, and the pooled C-index was 0.82 (95%CI: 0.81, 0.83), with no sensitivity or specificity. The most common algorithm of ML prediction models for out-of-hospital mortality was the LR (*N* = 10) [C-index: 0.84 (95%CI: 0.81, 0.88); sensitivity: 0.70 to 0.77; specificity: 0.61 to 0.75] (Tables 1 and 2).

##### Mature Clinically Recommended Tools

In the training set, there were 18 clinical scoring tools and some tools externally validated in large-scale cohort studies for predicting out-of-hospital mortality, and the pooled C-index was 0.817 (95%CI: 0.789, 0.845). The sensitivity and specificity of 7 tools were reported, and the pooled sensitivity and specificity were 0.75 (95%CI: 0.68, 0.80) and 0.79 (95%CI: 0.73, 0.84), respectively. In the verification set, there were only 4 tools predicting out-of-hospital mortality, and the pooled C-index was 0.813 (95%CI: 0.789, 0.836), with no sensitivity or specificity.

## Discussion

### Main findings

Our study is the first of its kind to systematically review the performance of ML in predicting the mortality of patients with TBI. According to the meta-analysis of 47 studies, ML models are highly accurate in predicting the mortality of patients with TBI. For instance, the C-index of most ML models such as the SVM, DT, LR, RF, and NN was greater than 0.8, especially the C-index of the NN, which was greater than 0.9. In addition, our studies also included some clinical scoring tools. The C-index of GCS predicting in-hospital mortality was 0.88 (95% CI: 0.85, 0.89), the sensitivity was 0.50 to 0.51, and the specificity was 0.90. Further, external validated tools in large-scale cohort studies were also included, such as CRASH core [C-index: 0.92 (95% CI: 0.89, 0.96); sensitivity: 0.73; specificity: 0.90] and IMPACT ct [C-index: 0.88 (95%CI: 0.84, 0.91); sensitivity: 0.80; specificity: 0.78]. In terms of in-hospital mortality, there was no huge difference in the overall prediction performance of ML and traditional scoring tools, but the prediction performance of the NN and batch normalization was outstanding. Regarding out-of-hospital mortality, the prediction performance of ML was slightly superior to traditional clinical scoring and some tools externally validated in large cohort studies. Recently published studies have revealed that there is a trend that the performance of the ML model is superior to that of LR, which may be related to the recent improvements in ML algorithms and the increasing popularity of special statistical software. The clinical decision support system should be constantly updated and improved, to meet the high requirements for risk prediction models in clinical practice, and further realize multidisciplinary collaborative decision-making as well as the benchmarking and monitoring of achievements. However, for predicting out-of-hospital mortality, LR is still the most frequently used model, which is easy to understand and realize owing to its simplicity, with no need for complex mathematical knowledge. In addition, LR can better interpret the result as well as the influence of variables on the result by coefficients, making it easy to analyze and understand. Furthermore, LR is easy to realize, because most programming languages provided corresponding bases and tools, making it easy to develop and apply.

### Clinical practice findings

In clinical practice, the initial assessment and treatment of acute TBI are still dependent on the GCS scale. GCS remains the most widely used scoring system for assessing the severity of TBI because it is fast and direct. Despite its utility, GCS also has important limitations, and cannot provide sufficient information, especially in the diagnosis of mild sub-clinical TBI and the prediction of TBI lineage. In the case of mild sub-clinical TBI, patients may have no obvious neurological symptoms, but GCS can only determine the score by evaluating the eye, oral, and limb reactions. Therefore, it may be not sensitive enough and may fail to detect mild TBI, especially mild cognitive and emotional disorders, which may affect the life quality and work capacity of patients. In addition, GCS cannot predict the recovery and long-term prognosis of patients. Although GCS can provide estimated information about the severity of brain injuries, it is still inaccurate to predict the recovery conditions and prognosis of patients by merely relying on the GCS score [60]. Although GCS can be used to predict in-hospital mortality, objectivity and accuracy are still poor, for instance, the speech components of intubated patients cannot be accurately assessed. In such cases, some doctors give the lowest possible score, while others infer the speech reaction score from other neurological manifestations [61]. Besides, GCS cannot detect subtle clinical changes in unconscious patients, such as abnormal brainstem reflexes and abnormal limb postures [62]. Add four score: therefore, we also pay attention to the comprehensive nonresponsiveness scale (four score) for predicting mortality in TBI. It is a new coma scale developed for the limitations of GCs, including eye opening (E), motor function (m), brainstem reflex (b) and respiratory mode (R). Compared with GCS, four score has similar mortality prediction ability, It seems that it can be used as an alternative to predict the early mortality of TBI patients in ICU [63]. Because it has some advantages, for example, in intubated patients, all components of the four score can be scored, but the GCS score cannot be scored [64]. However, its advantages are not obvious. Evidence based medicine evidence shows that the GCS and four scores are of equal value in predicting in-hospital mortality and adverse outcomes. The similar performance of these scores in the evaluation of TBI patients allows medical staff to choose to use any of them according to the situation at hand [65]. And we found that there is no comparative study between ML and the total nonresponsiveness scale in TBI.

In contrast, ML models use a large amount of real data for training and prediction, have wider coverage, contain more variables, and can reveal potential correlations and rules. The development of clinical scoring tools is inevitably subject to the subjective influence of investigators, and some variables may be ignored or over-considered. Additionally, ML models can carry out accurate and detailed predictions based on manifestations and characteristics of cases, thereby avoiding clinical scoring tools’ problem that their results may be too vague and too generalized. Furthermore, ML models can not only learn and optimize prediction algorithms adaptively, thus improving prediction accuracy, but also can implement personalized prediction and design of therapeutic plans based on the characteristics and historical data on patients [66]. However, clinical scoring tools tend to recommend templates and standardized therapeutic plans, which cannot satisfy the needs of different patients. Finally, ML prediction models can process various types of data, including imageology, laboratory, and biochemical information, to help doctors comprehensively evaluate the conditions of patients, while clinical scoring tools usually focus only on a few important indexes, so some implicit influencing factors may be ignored.

ML models used for medical purposes have their respective specificity and applicability, and different models are required for different diseases and clinical problems [67, 68]. However, due to the lack of studies with large sample sizes and in absence of a clear consensus, it is difficult to select a suitable model. Therefore, it is necessary to comprehensively analyze different algorithms and hyper-parameters to find the optimal model [69], and meanwhile consider the precision, complexity and training time of the model. Finally, the performance of the selected model can be assessed by the techniques such as cross-verification, so as to ensure its best efficiency in practical application [70]. ML algorithms make some hypotheses for the relationship between predictors and the target variables, which is called learning deviation, and introduce deviation into the model. The hypothesis made by ML algorithms indicates that some algorithms are more suitable for certain datasets than other algorithms [71]. Therefore, the degree of improvement by ML and the clinical influence are still uncertain. Nonetheless, it is certain that the big data analysis of ML is highly effective for the absorption and assessment of massive complex medical healthcare data. In addition, among the variables we calculated, the GCS score, age, CT classification, pupil, and glucose assay ranked top. GCS, as a clinical scoring tool, has become a variable in the ML model, which is not beyond our expectations, because it contains some key indexes for assessing the prognosis such as language, awareness, and action [72]. Interestingly, we have found the significance of CT classification in predicting the prognosis of TBI, which conforms to the clinical application of CT imaging to intuitively assess TBI of patients, predict the prognosis, help determine therapeutic strategies, and provide information for preparing such rehabilitation plan [73]. However, given the huge amount of CT imaging data, hundreds to thousands of images can be obtained per scanning, with complex data, including many different tissues and structures. Sometimes, artifacts may also affect the quality of images. Therefore, it is difficult to make progress in the traditional studies on CT data and clinical disease changes. Nevertheless, ML can automatically process a large number of clinical CT imaging data and extract useful characteristics, with no need for manual intervention, significantly lowering the time and costs required for clinical CT imaging data processing and analysis, and improving work efficiency. ML algorithms can rapidly and accurately identify and position key organs, structures, and anomalies, and further help doctors make more accurate and scientific medical decisions. In addition, it can also make personalized therapeutic plans, allowing more accurate treatment for patients and improving therapeutic effects [74]. In the future, studies on ML and CT imaging will be further implemented, and ML will be applied in a more extensive range. In this process, larger cohort studies are required to support its clinical application. Meanwhile, interdisciplinary cooperation becomes more and more important, including the cooperation of experts from the medicine, computer science, and mathematical sectors, to speed up the application and development of ML in CT imaging.

Our advantage is that we have conducted a comprehensive retrieval strategy and a throughout analysis. We have included any models for predicting the mortality of TBI, including not only ML models but also traditional tools externally validated in large cohort studies and traditional scoring tools. The most important advantage of ML algorithms is to more effectively handle missing data, and implement more complex calculations. It can perform a non-linear prediction of outcomes, without relying on the data distribution hypothesis, such as the independence and multiple collinearities of observation. Owing to outstanding model identification capacity, ML can help clinicians make decisions, and it has also been demonstrated to have an outstanding performance in identifying endocrine diseases [75] and predicting mortality of inflammatory diseases, etc. [76] .

Our study also has certain limitations. Firstly, the study only included the articles written in English, so it may fail to fully consider the relevant literature in other languages. Secondly, data conversion was used in data extraction, which may result in certain deviations. The AUC curve, sensitivity, and specificity were not reported in some of the included studies. Finally, although the meta-analysis has fully summarized the characteristics and trends of relevant literature, more refined explorations are still required, for instance, on the prediction capacity of ML for TBI mortality of different patient groups in different regions, which can provide a clear understanding of the actual feasibility of ML in predicting TBI mortality.

## Conclusion

According to the systematic review and meta-analysis, ML models can predict mortality of TBI, with an excellent discriminative ability in case-control studies. The key factors related to the model performance include the accepted clinical variables of TBI and the use of CT imaging. Although a single model is often superior to traditional scoring tools, the assessment of the summary performance is restricted by the heterogeneity of studies. Therefore, it is necessary to develop a standardized report guideline on ML for TBI. Currently, ML models are rarely used clinically, so it is in urgent demand for determining their clinical influence on different patient groups, to ensure their universality and apply the theoretical knowledge to clinical practices.

## Supplementary Information

**Additional file 1: Table S1.** Literature search strategy.

**Additional file 2: Table S2.** Predictors.

## Data Availability

All data generated or analyzed during this study are included in this published article (and its Supplementary Information files).
