# Supplementary material for: Prediction performance of the machine learning model in predicting mortality risk in patients with traumatic brain injuries: a systematic review and meta-analysis
Source: BMC Med Inform Decis Mak. 2023 Jul 29;23:142. doi: 10.1186/s12911-023-02247-8 (PMC10385965; doi:10.1186/s12911-023-02247-8)
Supplement: Supplementary file 1 — Additional file 1: Table S1. Literature search strategy. [file 12911_2023_2247_MOESM1_ESM.docx]

# Table S1 Literature search strategy

**1.Pubmed**

| Search number | Query | Results |
| --- | --- | --- |
| #1 | Brain Injuries, Traumatic[MeSH Terms] | 22578 |
| #2 | "Brain Contusion*"[Title/Abstract] OR "brain system trauma"[Title/Abstract] OR "Brain Trauma*"[Title/Abstract] OR "Cerebellar Contusion*"[Title/Abstract] OR "Cerebral Concussion*"[Title/Abstract] OR "Cerebral Contusion*"[Title/Abstract] OR "cerebral trauma"[Title/Abstract] OR "cerebrovascular trauma"[Title/Abstract] OR "Commotio Cerebri"[Title/Abstract] OR "Contusio Cerebri"[Title/Abstract] OR "Cortical Contusion*"[Title/Abstract] OR "Intermediate Concussion*"[Title/Abstract] OR "Mild Concussion*"[Title/Abstract] OR "mild traumatic brain injury"[Title/Abstract] OR "organic cerebral trauma"[Title/Abstract] OR "posttraumatic encephalopathy"[Title/Abstract] OR "Severe Concussion*"[Title/Abstract] OR "traumatic brain injuries"[Title/Abstract] OR "traumatic brain injury"[Title/Abstract] OR "traumatic brain lesion"[Title/Abstract] OR "traumatic cerebral lesion"[Title/Abstract] OR "Traumatic Encephalopathies"[Title/Abstract] OR "traumatic encephalopathy"[Title/Abstract] | 51082 |
| #3 | (Brain Injuries, Traumatic[MeSH Terms]) OR ("Brain Contusion*"[Title/Abstract] OR "brain system trauma"[Title/Abstract] OR "Brain Trauma*"[Title/Abstract] OR "Cerebellar Contusion*"[Title/Abstract] OR "Cerebral Concussion*"[Title/Abstract] OR "Cerebral Contusion*"[Title/Abstract] OR "cerebral trauma"[Title/Abstract] OR "cerebrovascular trauma"[Title/Abstract] OR "Commotio Cerebri"[Title/Abstract] OR "Contusio Cerebri"[Title/Abstract] OR "Cortical Contusion*"[Title/Abstract] OR "Intermediate Concussion*"[Title/Abstract] OR "Mild Concussion*"[Title/Abstract] OR "mild traumatic brain injury"[Title/Abstract] OR "organic cerebral trauma"[Title/Abstract] OR "posttraumatic encephalopathy"[Title/Abstract] OR "Severe Concussion*"[Title/Abstract] OR "traumatic brain injuries"[Title/Abstract] OR "traumatic brain injury"[Title/Abstract] OR "traumatic brain lesion"[Title/Abstract] OR "traumatic cerebral lesion"[Title/Abstract] OR "Traumatic Encephalopathies"[Title/Abstract] OR "traumatic encephalopathy"[Title/Abstract]) | 58274 |
| #4 | machine learning[MeSH Terms] | 51,458 |
| #5 | " machine learning"[Title/Abstract] OR " Transfer Learning"[Title/Abstract] OR "Deep learning"[Title/Abstract] OR "Ensemble Learning"[Title/Abstract] OR "artificial intelligence"[Title/Abstract] OR "Prediction model"[Title/Abstract] OR "random forest"[Title/Abstract] OR "neural network"[Title/Abstract] OR "neural networks"[Title/Abstract] OR "CNN"[Title/Abstract] OR "Support vector machine"[Title/Abstract] OR "SVM"[Title/Abstract] OR "Gradient Boosting Machine"[Title/Abstract] OR "GBM"[Title/Abstract] OR "Nomogram"[Title/Abstract] OR "XGBoost"[Title/Abstract] OR "Adaboost"[Title/Abstract] OR "Decision tree"[Title/Abstract] OR "ResNet-50"[Title/Abstract] OR "ResNet"[Title/Abstract] OR "Radiomics"[Title/Abstract] OR "Radiomic"[Title/Abstract] OR "Fine-Gray"[Title/Abstract] OR "Competitive Risk Model"[Title/Abstract] OR "Naive Bayesian"[Title/Abstract] OR "Risk Prediction"[Title/Abstract] OR "Risk-Prediction"[Title/Abstract] | 269457 |
| #6 | (machine learning[MeSH Terms]) OR (" machine learning"[Title/Abstract] OR " Transfer Learning"[Title/Abstract] OR "Deep learning"[Title/Abstract] OR "Ensemble Learning"[Title/Abstract] OR "artificial intelligence"[Title/Abstract] OR "Prediction model"[Title/Abstract] OR "random forest"[Title/Abstract] OR "neural network"[Title/Abstract] OR "neural networks"[Title/Abstract] OR "CNN"[Title/Abstract] OR "Support vector machine"[Title/Abstract] OR "SVM"[Title/Abstract] OR "Gradient Boosting Machine"[Title/Abstract] OR "GBM"[Title/Abstract] OR "Nomogram"[Title/Abstract] OR "XGBoost"[Title/Abstract] OR "Adaboost"[Title/Abstract] OR "Decision tree"[Title/Abstract] OR "ResNet-50"[Title/Abstract] OR "ResNet"[Title/Abstract] OR "Radiomics"[Title/Abstract] OR "Radiomic"[Title/Abstract] OR "Fine-Gray"[Title/Abstract] OR "Competitive Risk Model"[Title/Abstract] OR "Naive Bayesian"[Title/Abstract] OR "Risk Prediction"[Title/Abstract] OR "Risk-Prediction"[Title/Abstract]) | 274422 |
| #7 | ((Brain Injuries, Traumatic[MeSH Terms]) OR ("Brain Contusion*"[Title/Abstract] OR "brain system trauma"[Title/Abstract] OR "Brain Trauma*"[Title/Abstract] OR "Cerebellar Contusion*"[Title/Abstract] OR "Cerebral Concussion*"[Title/Abstract] OR "Cerebral Contusion*"[Title/Abstract] OR "cerebral trauma"[Title/Abstract] OR "cerebrovascular trauma"[Title/Abstract] OR "Commotio Cerebri"[Title/Abstract] OR "Contusio Cerebri"[Title/Abstract] OR "Cortical Contusion*"[Title/Abstract] OR "Intermediate Concussion*"[Title/Abstract] OR "Mild Concussion*"[Title/Abstract] OR "mild traumatic brain injury"[Title/Abstract] OR "organic cerebral trauma"[Title/Abstract] OR "posttraumatic encephalopathy"[Title/Abstract] OR "Severe Concussion*"[Title/Abstract] OR "traumatic brain injuries"[Title/Abstract] OR "traumatic brain injury"[Title/Abstract] OR "traumatic brain lesion"[Title/Abstract] OR "traumatic cerebral lesion"[Title/Abstract] OR "Traumatic Encephalopathies"[Title/Abstract] OR "traumatic encephalopathy"[Title/Abstract])) AND ((machine learning[MeSH Terms]) OR (" machine learning"[Title/Abstract] OR " Transfer Learning"[Title/Abstract] OR "Deep learning"[Title/Abstract] OR "Ensemble Learning"[Title/Abstract] OR "artificial intelligence"[Title/Abstract] OR "Prediction model"[Title/Abstract] OR "random forest"[Title/Abstract] OR "neural network"[Title/Abstract] OR "neural networks"[Title/Abstract] OR "CNN"[Title/Abstract] OR "Support vector machine"[Title/Abstract] OR "SVM"[Title/Abstract] OR "Gradient Boosting Machine"[Title/Abstract] OR "GBM"[Title/Abstract] OR "Nomogram"[Title/Abstract] OR "XGBoost"[Title/Abstract] OR "Adaboost"[Title/Abstract] OR "Decision tree"[Title/Abstract] OR "ResNet-50"[Title/Abstract] OR "ResNet"[Title/Abstract] OR "Radiomics"[Title/Abstract] OR "Radiomic"[Title/Abstract] OR "Fine-Gray"[Title/Abstract] OR "Competitive Risk Model"[Title/Abstract] OR "Naive Bayesian"[Title/Abstract] OR "Risk Prediction"[Title/Abstract] OR "Risk-Prediction"[Title/Abstract])) | 771 |

**2.Cochrane**

| Search number | Query | Results |
| --- | --- | --- |
| #1 | MeSH descriptor: [Machine Learning] explode all trees | 274 |
| #2 | (‘machine learning’ OR 'Transfer Learning' OR 'Deep learning' OR 'Ensemble Learning' OR 'artificial intelligence' OR 'Prediction model' OR 'random forest' OR 'neural network' OR 'neural networks' OR 'CNN' OR 'Support vector machine' OR 'SVM' OR 'Gradient Boosting Machine' OR 'GBM' OR 'Nomogram' OR 'XGBoost' OR 'Adaboost' OR 'Decision tree' OR 'ResNet-50' OR 'ResNet' OR 'Radiomics' OR 'Radiomic' OR 'Fine-Gray' OR 'Competitive Risk Model' OR 'Naive Bayesian' OR 'Risk Prediction' OR 'Risk-Prediction'):ti,ab,kw | 17993 |
| #3 | MeSH descriptor: [Brain Injuries, Traumatic] explode all trees | 1113 |
| #4 | ( 'Brain Contusion*' OR 'brain system trauma' OR 'Brain Trauma*' OR 'Cerebellar Contusion*' OR 'Cerebral Concussion*' OR 'Cerebral Contusion*' OR 'cerebral trauma' OR 'cerebrovascular trauma' OR 'Commotio Cerebri' OR 'Contusio Cerebri' OR 'Cortical Contusion*' OR 'Intermediate Concussion*' OR 'Mild Concussion*' OR 'mild traumatic brain injury' OR 'organic cerebral trauma' OR 'posttraumatic encephalopathy' OR 'Severe Concussion*' OR 'traumatic brain injuries' OR 'traumatic brain injury' OR 'traumatic brain lesion' OR 'traumatic cerebral lesion' OR 'Traumatic Encephalopathies' OR 'traumatic encephalopathy' ):ti,ab,kw | 6312 |
| #5 | (#1 OR #2)AND(#3 OR #4) | 179 |

**3.Embase**

| Search number | Query | Results |
| --- | --- | --- |
| #1 | 'traumatic brain injury'/exp | 62494 |
| #2 | 'brain contusion*':ti,ab,kw OR 'brain system trauma':ti,ab,kw OR 'brain trauma*':ti,ab,kw OR 'cerebellar contusion*':ti,ab,kw OR 'cerebral concussion*':ti,ab,kw OR 'cerebral contusion*':ti,ab,kw OR 'cerebral trauma':ti,ab,kw OR 'cerebrovascular trauma':ti,ab,kw OR 'commotio cerebri':ti,ab,kw OR 'contusio cerebri':ti,ab,kw OR 'cortical contusion*':ti,ab,kw OR 'intermediate concussion*':ti,ab,kw OR 'mild concussion*':ti,ab,kw OR 'mild traumatic brain injury':ti,ab,kw OR 'organic cerebral trauma':ti,ab,kw OR 'posttraumatic encephalopathy':ti,ab,kw OR 'severe concussion*':ti,ab,kw OR 'traumatic brain injuries':ti,ab,kw OR 'traumatic brain injury':ti,ab,kw OR 'traumatic brain lesion':ti,ab,kw OR 'traumatic cerebral lesion':ti,ab,kw OR 'traumatic encephalopathies':ti,ab,kw OR 'traumatic encephalopathy':ti,ab,kw | 73490 |
| #3 | #1 OR #2 | 85553 |
| #4 | 'machine learning'/exp | 348948 |
| #5 | 'machine learning':ti,ab,kw OR 'transfer learning':ti,ab,kw OR 'deep learning':ti,ab,kw OR 'ensemble learning':ti,ab,kw OR 'artificial intelligence':ti,ab,kw OR 'prediction model':ti,ab,kw OR 'random forest':ti,ab,kw OR 'neural network':ti,ab,kw OR 'neural networks':ti,ab,kw OR 'cnn':ti,ab,kw OR 'support vector machine':ti,ab,kw OR 'svm':ti,ab,kw OR 'gradient boosting machine':ti,ab,kw OR 'gbm':ti,ab,kw OR 'nomogram':ti,ab,kw OR 'xgboost':ti,ab,kw OR 'adaboost':ti,ab,kw OR 'decision tree':ti,ab,kw OR 'resnet-50':ti,ab,kw OR 'resnet':ti,ab,kw OR 'radiomics':ti,ab,kw OR 'radiomic':ti,ab,kw OR 'fine-gray':ti,ab,kw OR 'competitive risk model':ti,ab,kw OR 'naive bayesian':ti,ab,kw OR 'risk prediction':ti,ab,kw OR 'risk-prediction':ti,ab,kw | 343584 |
| #6 | #3 OR #4 | 523733 |
| #7 | #5 AND #6 | 1426 |

**4.Web of science**

| Search number | Query | Results |
| --- | --- | --- |
| #1 | TS=(traumatic brain injury or Brain Contusion* or brain system trauma or Brain Trauma* or Cerebellar Contusion* or Cerebral Concussion* or Cerebral Contusion* or cerebral trauma or cerebrovascular trauma or Commotio Cerebri or Contusio Cerebri or Cortical Contusion* or Intermediate Concussion* or Mild Concussion* or mild traumatic brain injury or organic cerebral trauma or posttraumatic encephalopathy or Severe Concussion* or traumatic brain injuries or traumatic brain injury or traumatic brain lesion or traumatic cerebral lesion or Traumatic Encephalopathies or traumatic encephalopathy) | 81557 |
| #2 | TS=(machine learning or Transfer Learning or Deep learning or Ensemble Learning or artificial intelligence or Prediction model or random forest or neural network or neural networks or CNN or Support vector machine or SVM or Gradient Boosting Machine or GBM or Nomogram or XGBoost or Adaboost or Decision tree or ResNet-50 or ResNet or Radiomics or Radiomic or Fine-Gray or Competitive Risk Model or Naive Bayesian or Risk Prediction or Risk-Prediction) | 1267379 |
| #3 | #2 AND #1 | 3060 |
